# Isolation and Characterization of Novel Human Parechovirus from Clinical Samples

**DOI:** 10.3201/eid1306.060896

**Published:** 2007-06

**Authors:** Kanako Watanabe, Masayasu Oie, Masaya Higuchi, Makoto Nishikawa, Masahiro Fujii

**Affiliations:** * Niigata University Graduate School of Medical and Dental Sciences, Niigata, Japan; † Niigata Prefectural Institute of Public Health and Environmental Sciences, Niigata, Japan

**Keywords:** Human parechovirus, subtype, HPeV-6, research

## Abstract

Using Vero cells, we isolated a virus (NII561-2000) from a cerebrospinal fluid specimen of a 1-year-old girl with Reye syndrome. The determined amino acid sequence of the virus indicated that the isolate was a human parechovirus (HPeV), a member of *Picornaviridae*. Neutralization test showed that the NII561-2000 virus had distinct antigenicity to HPeV-1, HPeV-2, and HPeV-3, and that the sequence was distinct from these types as well as from HPeV-4 and HPeV-5. Thus, we propose the virus (NII561-2000) as the prototype of HPeV-6. We isolated 10 NII561-2000–related viruses, 14 HPeV-1, 16 HPeV-3, and 1 HPeV-4 of 41 HPeVs from various clinical samples collected in Niigata, Japan. Clinical symptoms of the persons infected with the NII561-2000–related viruses were infectious gastroenteritis, rash, upper respiratory tract infection, and paralysis, in addition to Reye syndrome in the 1-year-old girl.

## Abstract 2

Identification of HPeV-6 will advance HPeV diagnosis and epidemiology.

Human parechovirus (HPeV) is a small, nonenveloped RNA virus with a single-stranded genome of positive polarity, ≈7.3 kb in length; it is a member of the *Picornaviridae* family (*1*–*3*). On the basis of serologic and genetic studies, HPeV has been found to have 5 types, HPeV-1, HPeV-2, HPeV-3, HPeV-4, and HPeV-5, with 76.0%–80.9% similarity at the nucleotide level and 84.7%–90.0% similarity at the amino acid level (*1*,*4*–*7*). HPeV infections are commonly observed in general populations. For example, ≈20% of healthy children in Finland have antibodies against HPeV-1, and the percentage is as high as 97% in adults (*8*). In addition, these viruses are frequently isolated from patients with various human diseases, such as gastroenteritis, encephalitis, flaccid paralysis, and respiratory infections, and they are thought to be associated with these diseases (*2*,*3*,*5*,*8*,*9*).

We report a 1-year-old girl who died with Reye syndrome, which is characterized as an acute, noninflammatory encephalopathy with hepatic dysfunction and fatty infiltration of the viscera; the syndrome is frequently associated with an antecedent viral infection, such as influenza or varicella (*10*–*12*). Inoculation of a cerebrospinal fluid (CSF) specimen from the patient into Vero cells identified a virus (NII561-2000) with similar properties to HPeVs. The nucleotide sequence of this virus showed it was closely related to HPeVs, especially HPeV-1, with 79.5% nucleotide and 90.7% amino acid (aa) similarities. Moreover, mutual neutralization assay showed that NII561-2000 has distinct antigenicity to HPeV-1, HPeV-2, and HPeV-3. In addition, the NII561-2000 virus was genetically distinct from HPeV-4 and HPeV-5. Thus, we propose that NII561-2000 is the prototype of HPeV-6.

## Materials and Methods

### Cell Lines and Culture Conditions

Eight adherent cell lines, MDCK, Caco-2, RD-18S, Vero, HeLa, HEp-2, LLC-MK2, and BSC-1, were used in our study. In brief, MDCK originated from a kidney of a normal adult cocker spaniel, Caco-2 was from a primary colorectal adenocarcinoma, RD-18S was from a rhabdomyosarcoma, Vero was from the kidney of a normal adult African green monkey, HeLa was from a cervical adenocarcinoma, HEp-2 was from an epidermoid carcinoma of the larynx, LLC-MK2 was from a kidney of a normal adult rhesus monkey, and BSC-1 was from a kidney of a normal adult African green monkey. These cell lines were cultured in Eagle minimum essential medium with 6 mmol/L L-glutamine, 1.1 g/L sodium bicarbonate, antimicrobial agents (0.2 g/L gentamicin, 0.25 g/L amphotericin B), and 8% fetal bovine serum at 37°C under 5% carbon dioxide.

### Virus Isolation and Purification

MDCK, Caco-2, RD-18S, Vero, HeLa, HEp-2, LLC-MK2, and BSC-1 cells were cultured on 24-well plates for 3 days. Then, these cells were inoculated with a CSF specimen (100 µL per well) from a patient with a diagnosis of Reye syndrome and cultured at 33°C for 2 weeks. To check for cytopathic effect (CPE), we examined the cells under a light microscope. To purify the virus particles, we mixed 900 mL culture fluid of Vero cells inoculated with the NII561-2000 virus with 20 g NaCl and 76 g polyethylene glycol #6,000 at 4°C overnight. Next, the sample was centrifuged at 5,000 rpm for 30 min, and the pellets were suspended in 4.5 mL of phosphate buffer (pH 7.2). After treatment with 4.5 mL of chloroform at 4°C for 5 min, the sample was centrifuged at 2,500 rpm for 20 min. The supernatant was centrifuged at 120,000 × *g* for 24 h in cesium chloride (CsCl) solution at an initial density of 1.34 g/mL, and the virion-containing fraction was collected and used for viral RNA isolation.

### Molecular Cloning

Double-stranded cDNA was synthesized from 5 µg of the viral RNA from the viruses purified by the CsCl density gradient ultracentrifugation method described above. The nucleotide sequences of the cDNAs from randomly picked-up bacterial colonies transfected with the cDNA-containing plasmids were determined by using the Big Dye sequencing kit (Applied Biosystems, Foster City, CA, USA). Two cDNAs isolated contained part of the NII561-2000 gene. To isolate the 3′ cDNA fragment of the isolated NII561-2000 cDNAs, the 3′ rapid amplification of cDNA ends (RACE) was performed by using the RNA PCR Kit (AMV) according to the instructions provided by the supplier (TaKaRa, Kyoto, Japan). The primers used for 3′ RACE were 35F-out (forward primer for the first PCR; 5′-GAT GCG GAA AAC TGC TGG ACA C-3′), 35F-in (forward primer for the second PCR; 5′-TGC CAA ATT TTT CTG CCC TAC TG-3′), and M13M4 (reverse primer for the first and second PCR; 5′-GTT TTC CCA GTC ACG AC-3′). Herculase Hotstart DNA Polymerase (Stratagene, La Jolla, CA, USA) was used. To isolate the 5′ cDNA fragment of the isolated NII561-2000, the cDNA fragment containing part of the 5′ untranslated region (UTR) and capsid precursor protein VP0 was amplified by PCR from the cDNA prepared from the NII561-2000 virus with degenerate primers corresponding to this region. The degenerate primers used were E23P1 (forward primer; 5′-CCG YAG GTA ACA AGW GAC AT-3′) (*5*) and 35R (reverse primer; 5′-TCT CAG CAC TAA TGA CCC TC-3′). To further extend the sequence information of 5′ UTR of the NII561-2000 virus, the 5′ RACE was performed with 5′ RACE System for Rapid Amplification of cDNA Ends (Version 2.0), by using the instructions provided by the supplier (Invitrogen, San Diego, CA, USA). The primers used for 5′ RACE were AAP (forward primer for the first PCR; 5′-GGC CAC GCG TCG ACT AGT ACG GGI IGG GII GGG IIG-3′), HPeV-GSP2 (reverse primer for the first PCR; 5′-AGA TGC ATC ATC TGC GAC TC-3′), UAP (forward primer for the second PCR; 5′-CUA CUA CUA CUA GGC CAC GCG TCG ACT AGT AC-3′), and HPeV-GSP (reverse primer for the second PCR; 5′GCC ATG TCT GCA ATG CTC TT-3′). *Taq* polymerase (Biotech International, Bentley, Western Australia, Australia) was used as a polymerase. Because 5′ RACE did not reach to the 5′ end of the NII561-2000 virus cDNA, the 5′ end cDNA fragment of the NII561-2000 virus was amplified from the NII561-2000 viral RNA by reverse transcription–PCR (RT-PCR). The primers used for RT-PCR were HPeV-head (forward primer; 5′-TTT GAA AGG GGT CTC CT-3′) and HPeV-mid (reverse primer; 5′-CAT AAG TTC CAC AAG CGT GG-3′ HPeV-head primer was designed as a conserved 5′ end sequence of the HPeVs 5′ UTR.

### Neutralization Test

Twenty-five microliters (100 median tissue culture doses; 50% tissue infective dose [TCID_50_]) of the indicated viruses and 25 µL of the serially diluted antisera (an initial 10-fold dilution and then 2-fold serial dilutions) were mixed in a 96-well plate and incubated at 37°C for 2 h. Then suspended Vero cells (100 µL/well) were added into these wells. Three to 10 days later the CPE of Vero cells was checked by light microscopy. To prepare the antiserum against the NII561-2000 virus, the virus particles grown in Vero cells were purified by CsCl centrifugation as described above. By using this purified virus, we prepared antiserum by Nippon Biotest Laboratory (Tokyo, Japan). In brief, the purified viruses were subcutaneously injected into rabbits 3× every 2 weeks. After we checked the antiviral titer by the Ouchterlony method, blood was collected from the vaccinated rabbits.

### Comparisons of VP0 Amino Acid Sequences of Clinical *i*solates

To determine the VP0 amino acid sequences of viruses isolated from clinical samples with cultured cell lines, we extracted the viral RNA from culture supernatant of cells injected with the clinical samples by using the High Pure Viral RNA Kit (Roche, Mannheim, Germany). cDNA was synthesized from 8 µL of the viral RNA by using 1 U Moloney murine leukemia virus reverse transcriptase (Invitrogen) and 20 U of recombinant RNAs in ribonuclease inhibitor (Promega, Madison, WI, USA). The 810-bp fragment containing part of 5′ UTR and VP0 was amplified by PCR, using 5 µL of cDNA in a 50-µL reaction mixture containing 50 mmol/L KCl, 10 mmol/L Tris/HCl (pH 8.5), 2.5 mmol/L MgCl_2_, 0.2 mmol/L of each deoxynucleoside triphosphates, 50 pmol of each primer, and 1 U of *Taq* polymerase (Biotech International). The amplification reaction consisted of 30 cycles at 95°C for 30 s, at 59°C for 30 s, and at 72°C for 1 min. The primer set used was E23P1 as a forward primer and HPV-N1 (5′-TAG GGG ATA CAT ARG TCR GCY T-3′ as a reverse primer (*5*).

### Phylogenic Tree Analysis

The bootstrap values were calculated from P1 amino acid sequences of parechoviruses with the CLUSTAL X software program (*13*), and the phylogenetic tree of these P1 amino acid sequences was constructed by using the neighbor-joining method. The nucleotide sequences of the following parechoviruses were obtained from GenBank: and their accession numbers were L02971 for HPeV-1 (Harris strain), AJ005695 for HPeV-2 (Williamson strain), AB084913 for HPeV-3(A308/99), AJ889918 for HPeV-3 (Can82853-01), DQ315670 for HPeV-4 (K251176-02), AM235750 for HPeV-4 (T75-4077), AF055846 for HPeV-5 (CT86-6760), AM235749 for HPeV-5 (T92-15), and AF327920 for Ljungan virus (LV). The GenBank/EMBL/DDBJ accession numbers of NII428-2000, NII2392-2001, NII2667-2001, NII2694-2001, NII2729-2001, and NII561-2000 are AB252577–AB252582.

## Results

### Virus Isolation

In January 2000, a 1-year-old girl with croup and high fever (39.6°C) was hospitalized in a regional general hospital in Niigata Prefecture, Japan. The infant died, and her condition was diagnosed as Reye syndrome after postmortem pathologic examination. To identify the pathogenic agent, we added a CSF specimen collected before death to 8 cell lines: MDCK, Caco-2, RD-18S, Vero, HeLa, HEp-2, LLC-MK2, and BSC-1 cells. Only Vero cells inoculated with the specimen exhibited a CPE. The CPE titer of the culture fluid was 10^5^–10^6^ TCID_50_ per 25 µL against Vero cells. Electron microscopic examination detected typical enteroviruslike virions in the culture fluid of the specimen (round, no envelop, ≈30 nm in diameter). Taken together, these results suggested that the CSF from the patient contained a virus, which we refer to here as NII561-2000.

### Physical and Antigenic Properties

Neutralization tests were performed to examine whether the NII561-2000 agent is related to known viruses. The NII561-2000 virus infection of Vero cells was not neutralized by a pool of enterovirus typing antisera or 3 HPeV typing antisera [(HPeV-1, HPeV-2, and HPeV-3) (A308/99)] (Table 1). Conversely, the rabbit antiserum to the NII561-2000 virus did not neutralize the infection of prototype strains of echovirus (serotypes 1–6, 9, 11–15, 17–21, 24–27, 29, 30, and 33), enteroviruses (serotypes 68 and 69), and 3 HPeVs, while it efficiently inhibited the infection of the NII561-2000 virus. These results suggest that the NII561-2000 agent is distinct from the examined known enteroviruses and HPeVs.

**Table 1:** Neutralization activities of anti-HPeV antibodies using Vero cells*

Antiserum	Virus			
	NII561-2000	HPeV-1	HPeV-2	HPeV-3
NII561-2000	160	10	<10	<10
HPeV-1†	<10	>1,280	<10	<10
HPeV-2	<10	<10	160	<10
HPeV-3	<10	<10	<10	>1,280

*HPeV, human parechovirus. †The HPeV strains used were Harris strain for HPeV-1, Williamson strain for HPeV-2, and A308/99 for HPeV-3.

We next examined the sensitivity of the virus to 5-iodo-2′-deoxyuridine (IUDR) (*14*). The NII561-2000 virus, treated with 10–4.5 µmol/L IUDR, was injected into Vero cells. The IUDR treatment did not affect the CPE in Vero cells, indicating that the virus has the RNA genome (data not shown). We next examined the acid-stability and thermostability of the NII561-2000 virus. The virus was treated at pH 3.0 for 3 h at room temperature, but the infectivity of the treated virus to Vero cells was little affected. Incubation of the virus at 50°C for 30 min reduced the infectivity, while incubation at 50°C for 1 h in the presence of 1 mol/L MgCl_2_ did not reduce the infectivity. These results indicate that the NII561-2000 virus has similar properties to human enteroviruses and HPeVs.

### Genetic Analysis of NII561-2000

To determine the nucleotide sequence of the NII561-2000 virus, the viral RNA was extracted from the purified virus. The partial nucleotide sequences of cDNA clones derived from this viral RNA were determined. BLAST (www.ncbi.nlm.nih.gov/blast) search identified that the nucleotide sequences of 2 isolated cDNAs showed high similarity with that of HPeV-2. By using these cDNAs as a starting material, the cDNAs containing the 5′ portion and 3′ portion of the NII561-2000 gene were isolated by RT-PCR with degenerated primers, 5′ RACE and 3′ RACE. The determined nucleotide sequence of NII561-2000 was 7,347 nt in length, excluding a poly (A) tract. Following a 709-nt 5′ UTR, a long open reading frame encoded a putative polyprotein precursor of 2,182 aa, which was followed by an 89-nt 3′ UTR. To verify the nucleotide sequence of the determined NII561-2000 genome, RT-PCR with a set of primers and RNA sample extracted from the virus-infected Vero cells was carried out. The nucleotide sequences of the NII561-2000 genome obtained by using this RT-PCR method were perfectly matched with those of the originally determined sequences (data not shown).

Phylogenetic tree analysis with complete P1 (VP0, VP3, and VP1) amino acid sequences showed that the NII561-2000 virus was most similar to HPeV-1/HPeV-2 (Figure, panel A). The nucleotide sequence and amino acid similarities of NII561-2000 virus to HPeV-1 were 79.5% and 90.7%, those with HPeV-2 were 77.1% and 87.3%, those with HPeV-3 (A308/99) were 76.7% and 85.9%, those with HPeV-4 were 77.1% and 88.2%, and those with HPeV-5 (CT86-6760) were 77.0% and 86.9%, respectively (Table 2). Thus, the NII561-2000 virus is the most similar to HPeV-1 at the amino acid level. The VP1 capsid gene of NII561-2000 was the most divergent (33.8% at nucleotide level) from that of HPeV-5 (CT86-6760).

**Figure:**
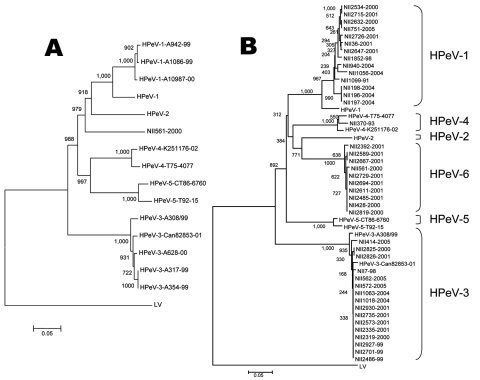
Phylogenetic tree analysis of the NII561-2000 virus and the NII561-2000–related viruses. A) The phylogenetic tree of P1 amino acid sequences was constructed as described in Materials and Methods. Bar shows a genetic distance of 0.05. The following amino acid sequences were obtained from GenBank: L02971 for HPeV-1 (Harris strain), AJ005695 for HPeV-2 (Williamson strain), AB084913 for HPeV-3 (A308/99), AJ889918 for HPeV-3 (Can82853-01), DQ315670 for HPeV-4 (K251176-02), AM235750 for HPeV-4 (T75-4077), AF055846 for HPeV-5 (CT86-6760), AM235749 for HPeV-5 (T92-15), and AF020541 for Ljungan virus (LV). B) Phylogenetic relationships among the NII561-2000 virus, the NII561-2000–related viruses, and other prototype HPeVs based on comparisons of amino acid similarities of capsid protein (the first 200 aa of VP0 sequences). The tree was constructed by the neighbor-joining method, using ClustalX. Bootstrap values are expressed in percentages after sampling 1,000×. Bar shows a genetic distance of 0.05.

**Table 2:** Comparisons of nucleotide and amino acid sequences among NII561-2000, HPeV-1, HPeV-2, HPeV-3, HPeV-4, and HPeV-5*

Sequence	% Nucleotide similarity (% amino acid similarity) with NII561-2000				
	HPeV-1†	HPeV-2	HPeV-3	HPeV-4	HPeV-5
5′UTR	85.3	87.3	86.9	88.5	86.9
VP0	73.6 (78.2)	76.0 (80.9)	70.6 (72.3)	72.6 (81.0)	73.1 (77.2)
VP3	74.4 (84.7)	73.0 (80.2)	67.0 (74.1)	70.9 (78.0)	68.7 (73.1)
VP1	71.7n(80.3)	67.3 (72.2)	71.1 (75.3)	70.0 (71.9)	66.2 (70.2)
2A	80.8 (90.7)	77.1 (88.0)	81.0 (85.3)	77.3 (91.2)	76.2 (88.7)
2B	78.6 (97.5)	77.8 (97.5)	82.0 (97.5)	79.8 (97.5)	78.7 (99.2)
2C	84.7 (97.3)	76.4 (86.9)	79.2 (92.7)	78.7 (92.4)	79.2 (93.6)
3A	85.7 (96.6)	77.4 (88.0)	78.0 (87.2)	75.5 (89.7)	78.6 (83.8)
3B	81.4 (80.0)	72.9 (80.0)	73.3 (75.0)	66.7 (85.0)	70.0 (85.0)
3C	84.2 (99.0)	82.7 (99.0)	82.3 (98.0)	82.5 (98.0)	80.5 (99.0)
3D	83.2 (95.9)	83.7 (95.5)	83.9 (95.7)	84.2 (96.8)	87.2 (97.0)
3′UTR	81.6	88.5	83.9	84.9	93.2
ORF	79.5 (90.7)	77.1 (87.3)	76.7 (85.9)	77.1 (88.2)	77.0 (86.9)

*HPeV, human parechovirus; UTR, untranslated region; ORF, open reading frame. †The HPeV strains used were Harris strain for HPeV-1, Williamson strain for HPeV-2, A308/99 for HPeV-3, K2511876–02 for HPeV-4, and CT86–6760 for HPeV-5.

HPeVs have 9 polyprotein cleavage sites: VP0/VP3, VP3/VP1, VP1/2A, 2A/2B, 2B/2C, 2C/3A, 3A/3B, 3B/3C, and 3C/3D (Table 3). Comparison of these polyprotein cleavage sites among 6 HPeVs showed that those in VP3/VP1, 2A/2B, 2B/2C, 2C/3A, 3A/3B, 3B/3C, and 3C/3D were conserved among all 6 HPeVs. The cleavage site in VP1/2A was identical among HPeV-1, HPeV-2, HPeV-4, HPeV-5, and NII561-2000 but not HPeV-3. The cleavage site in VP0/VP3 of NII561-2000 was identical to that of HPeV-3 but not to those of others.

**Table 3:** Amino acid sequences of protein cleavage sites of HPeVs

Virus	VP0/VP3	VP3/VP1	VP1/2A	2A/2B	2B/2C	2C/3A	3A/3B	3B/3C	3C/3D
NII561-2000	N/G	Q/N	Q/S	Q/G	Q/G	Q/T	E/R	Q/R	Q/G
HPeV-1*	N/A	Q/N	Q/S	Q/G	Q/G	Q/T	E/R	Q/R	Q/G
HPeV-2	T/A	Q/N	Q/S	Q/G	Q/G	Q/T	E/R	Q/R	Q/G
HPeV-3	N/G	Q/N	E/S	Q/G	Q/G	Q/T	E/R	Q/R	Q/G
HPeV-4	N/N	Q/N	Q/S	Q/G	Q/G	Q/T	E/R	Q/R	Q/G
HPeV-5	N/S	Q/N	Q/S	Q/G	Q/G	Q/T	E/R	Q/R	Q/G

*The human parechovirus (HPeV) strains used were Harris strain for HPeV-1, Williamson strain for HPeV-2, A308/99 for HPeV-3, K2511876–02 for HPeV-4, and CT86–6760 for HPeV-5.

The NII561-2000 virus, HPeV-1, HPeV-2, HPeV-4, and HPeV-5, but not HPeV-3, had an RGD (arginine-glycine-aspartic acid) motif at the C terminus of VP1 (*15*). The RGD motif may be used for an entry receptor of these HPeVs to attach, penetrate, or both, into host cells. Mutant HPeV-1 viruses with 2 aa deletions in the RGD motif showed little infectivity, while an RGD-to-RGE (arginine-glycine-glutamic acid) change showed reduced infectivity, and the resultant viruses possessed a rescued RGD. In addition, mutations at the +1 and +2 positions downstream from the RGD motif produced small virus–inducing plaques, and an M-to-P change at +1 position was lethal (*15*). The amino acids (+1 and +2 positions) downstream from the RGD motif of NII561-2000 were identical to those of HPeV-1.

The N terminal ends of VP4 of many picornaviruses are myristoylated, and they have a consensus myristoylation motif (*16*). HPeVs, including NII561-2000, lacked a myristoylation motif in the corresponding VP0 sequence. Myristoylation of capsid proteins is suggested to play a role in virion assembly. Thus, the virion assembly mechanism of HPeVs, including NII561-2000, might be distinct from other picornaviruses, including poliovirus.

### Isolation of HPeVs from Clinical Samples

By using cultured cells (Caco-2, RD-18S, Vero, HeLa, HEp-2, LLC-MK2, and BSC-1), we have isolated 8,195 CPE-inducing agents from 13,656 clinical samples (stool, throat swab, and CSF) collected between 1991 and 2005, at Niigata, Japan (Table 4, Table 5). The CPE morphologic features and CPE-inducing cell types suggested that 1,521 isolates are likely to be enteroviruses, HPeVs, rhinoviruses, or other viruses, and the others are likely to be influenza viruses or adenoviruses. Neutralization tests that used antisera against enteroviruses and HPeV-1 showed that 1,365 were enteroviruses and 12 were HPeV-1, and the remaining 144 viruses were not neutralized by these agents. RT-PCR that used degenerate primers against HPeVs, enteroviruses, and rhinoviruses identified 29 HPeVs, 72 enteroviruses, and 26 rhinoviruses, respectively. The remaining 17 viruses were not identified by these methods. Thus, a total of 41 HPeVs were isolated from the samples collected in Niigata.

**Table 4:** Numbers of isolated HPeVs in 1991–2005 at Niigata, Japan*

Year	HPeV subtype				No. isolated HPeVs	No. examined samples	No. isolated viruses
	Type 1	Type 3	Type 4	Type 6			
1991	1	0	0	0	1	303	255
1992	0	0	0	0	0	142	53
1993	0	0	1	0	1	166	50
1994	0	0	0	0	0	151	55
1995	0	0	0	0	0	167	116
1996	0	0	0	0	0	133	54
1997	0	0	0	0	0	277	84
1998	1	1	0	0	2	1,607	1,117
1999	0	3	0	0	3	2,118	1,203
2000	2	2	0	3	7	1,974	1,127
2001	4	5	0	7	16	1,699	989
2002	0	0	0	0	0	2,783	1,787
2003	0	0	0	0	0	986	597
2004	5	2	0	0	7	584	329
2005	1	3	0	0	4	566	379
Total	14	16	1	10	41	13,656	8,195

*HPeV, human parechovirus.

**Table 5:** Diseases associated with HPeVs isolated 1991–2005 in Niigata, Japan*

Strain	HPeV subtype	Specimen	Clinical symptom	Sex	Age, y	Cell line(s)
NII1099-91	1	Stool	Gastroenteritis	F	1	BSC-1
NII1852-98	1	Throat swab	Hand-foot-mouth disease	M	<1	CaCo2, RD-18S
NII2534-2000	1	Stool	Fever of unknown origin	M	<1	CaCo2
NII2632-2000	1	Stool	Gastroenteritis	M	1	RD-18S, Vero

NII36-2001	1	Stool	Gastroenteritis	M	1	BSC-1
NII2647-2001	1	Throat swab	Upper respiratory tract infection	M	1	RD-18S
NII2715-2001	1	Stool	Gastroenteritis	M	<1	CaCo2, RD-18S
NII2726-2001	1	Stool	Gastroenteritis	F	1	CaCo2
NII196-2004	1	Stool	Gastroenteritis	M	9	LLC-MK2
NII197-2004	1	Stool	Gastroenteritis	M	<1	LLC-MK2
NII198-2004	1	Stool	Gastroenteritis	M	<1	LLC-MK2
NII940-2004	1	Throat swab	Bronchitis	M	<1	CaCo2, RD-18S, Vero
NII1056-2004	1	Stool	Gastroenteritis	F	<1	LLC-MK2
NII751-2005	1	Stool	Gastroenteritis	M	<1	CaCo2
NII7-98	3	Stool	Gastroenteritis	F	1	BSC-1
NII2486-99	3	Stool	Rash	F	<1	Vero
NII2701-99	3	Throat swab	Rash	F	1	Vero
NII2927-99	3	Stool	Aseptic meningitis	M	<1	Vero
NII2319-2000	3	Stool	Upper respiratory tract infection	M	<1	BSC-1
NII2825-2000	3	Throat swab	Upper respiratory tract infection	M	1	Vero, LLC-MK2
NII2335-2001	3	Throat swab	Rash	F	<1	Vero, LLC-MK2
NII2573-2001	3	Throat swab	Rash	F	<1	Vero, LLC-MK2
NII2735-2001	3	Stool	Aseptic meningitis	F	<1	Vero, LLC-MK2
NII2826-2001	3	Throat swab	Upper respiratory tract infection	M	<1	Vero, LLC-MK2
NII2930-2001	3	Throat swab	Myositis	M	8	CaCo2, Vero
NII1018-2004	3	CSF	Fever of unknown origin	F	<1	Vero, LLC-MK2
NII1063-2004	3	Stool	Gastroenteritis	M	<1	CaCo2, LLC-MK2
NII414-2005	3	Throat swab	Influenzalike illness	M	4	Vero, LLC-MK2
NII562-2005	3	Throat swab	Influenzalike illness	F	35	LLC-MK2
NII572-2005	3	Throat swab	Influenzalike illness	M	5	Vero, LLC-MK2
NII370-93	4	Stool	Lymphadenitis	M	5	RD-18S, Vero, LLC-MK2
NII428-2000	6	Stool	Gastroenteritis	F	1	Vero
NII561-2000	6	CSF	Reye syndrome	F	1	Vero
NII2819-2000	6	Throat swab	Rash	M	2	BSC-1
NII2392-2001	6	Stool	Flaccid paralysis	M	1	CaCo2
NII2485-2001	6	Throat swab	Upper respiratory tract infection	F	<1	RD-18S
NII2589-2001	6	Throat swab	Gastroenteritis	M	8	RD-18S
NII2611-2001	6	Stool	Gastroenteritis	F	3	CaCo2, Vero
NII2667-2001	6	Stool	Gastroenteritis	M	6	HeLa
NII2694-2001	6	Throat swab	Rash	M	2	CaCo2, RD-18S
NII2729-2001	6	Stool	Gastroenteritis	F	2	BSC-1

*HPeV, human parechovirus; CSF, cerebrospinal fluid.

Phylogenetic tree analysis showed 14 HPeV-1, 16 HPeV-3, and 1 HPeV-4, but no HPeV-2 and HPeV-5 among the examined 41 HPeVs. In addition, 10 viruses, including NII561-2000, formed a distinctive tree from those of the other HPeVs (Figure, panel B). Of note, in our search from 1991 through 2005, the NII561-2000–related viruses were isolated only in 2000 (3 cases) and 2001 (7 cases); HPeV-1 were isolated in 1991, 1998, 2001, 2004, and 2005; HPeV-3 were isolated in 1998, 1999, 2000, 2001, 2004, and 2005; and HPeV-4 was isolated only in 1993 (Table 4). The clinical symptoms of the patients infected with NII561-2000–related viruses were gastroenteritis, respiratory symptoms, rash, and flaccid paralysis in addition to Reye syndrome (Table 5), and these disease categories were similar to those of other HPeVs (*2*,*3*).

## Discussion

In this study, we isolated a novel HPeV (NII561-2000) from a 1-year-old girl with Reye syndrome and determined the nucleotide sequence. Nucleotide sequence analysis and mutual neutralization test indicated that the NII561-2000 virus was distinct from 5 known HPeVs (Figure, panel A) (*17*). Thus, we propose that the NII561-2000 virus is the prototype of HPeV-6.

The NII561-2000 virus was originally isolated from a patient with Reye syndrome, an acute noninflammatory encephalopathy characterized by an antecedent viral infection, such as influenza or varicella (*10*–*12*). The significance of the NII561-2000 virus in this syndrome is not clear, because our samples did not include any other samples from this patient. We also isolated 9 NII561-2000–related viruses from clinical samples collected from other patients. The clinical symptoms of the persons infected with NII561-2000–related viruses were gastroenteritis, upper respiratory tract infection, rash, and flaccid paralysis. These disease categories of the NII561-2000–related virus are similar to those of other HPeVs, but the pathologic roles of the NII561-2000 viruses in these diseases, including Reye syndrome, need further etiologic and biologic studies.

In our search at Niigata from 1991 through 2005, the NII561-2000 and related viruses were isolated only in 2 consecutive years (2000 and 2001), HPeV-1 were isolated in 5 years, and HPeV-3 were isolated in 6 years. The genetic variations of these NII561-2000–related viruses were small (Figure, panel B). These results suggest that a 2-year outbreak of NII561-2000–related virus may have occurred in Niigata, Japan.

Thirteen of 16 HPeV-3 isolated in Niigata were from the patients <3 years of age, consistent with the previous report (*18*). Sepsislike illness and central nervous system involvement were more frequently reported in children infected with HPeV-3 than HPeV-1 (*18*). Consistent with those findings, the HPeV-3 infections in Niigata included 2 aseptic meningitis patients, whereas no such illness was associated with HPeV-1.

HPeV-4 was isolated from a 5-year-old patient with lymphadenitis in Niigata (Table 5). Thus, this virus is prevalent and is likely to be pathogenic in at least 3 countries. The diseases associated with HPeV-4 in the Netherlands and the United States were fever and TORCH (toxoplasmosis; other infections; namely, hepatitis B, syphilis, herpes zoster, rubella, cytomegalovirus, and herpes simplex virus infections) (*6*,*19*). Further analysis is required to establish an association of HPeV-4 with these diseases.

The degenerate primer set we used here was originally developed by Ito et al. (*5*) to amplify 3 known HPeV cDNAs. Here, we successfully amplified the NII561-2000 viral cDNA and HPeV-4 from culture supernatants of the infected cells, indicating that this primer set can amplify the cDNA fragments of at least 5 HPeVs from culture supernatants of infected cells. Thus, this primer set is a useful tool to determine the subtypes of HPeVs.
